# The Observable Representation

**DOI:** 10.3390/e21030310

**Published:** 2019-03-21

**Authors:** L. S. Schulman

**Affiliations:** Physics Department, Clarkson University, Potsdam, NY 13699-5820, USA; schulman@clarkson.edu

**Keywords:** observable representation, random walk, phase transition, spin-glass, foraging, linguistics, stochastic matrix, spectral analysis

## Abstract

The observable representation (OR) is an embedding of the space on which a stochastic dynamics is taking place into a low dimensional Euclidean space. The most significant feature of the OR is that it respects the dynamics. Examples are given in several areas: the definition of a phase transition (including metastable phases), random walks in which the OR recovers the original space, complex systems, systems in which the number of extrema exceed convenient viewing capacity, and systems in which successful features are displayed, but without the support of known theorems.

## 1. Introduction 

The observable representation (OR) is an embedding of the space on which a stochastic dynamics is taking place into Euclidean *n*-space. The value of the integer *n* is determined by the dynamics as well as by the user. The most significant feature of the observable representation is that it respects the dynamics. Thus points close to one another in this embedding tend to be dynamically related. This often allows an appreciation of what’s going on and insight into the relation of points to one  another.

Much (but not all) of the work described here was done in collaboration with Bernard Gaveau at the University of Paris VI. Other collaborators are thanked in the acknowledgements.

In this article the observable representation will be abbreviated OR. The word “observable” is meant to bring to mind the slowest varying quantities, which correspond to the observables of a system [1].

As far as I know, this method is conceptually different from principal component analysis [2,3], although both methods allow visibility of relations among elements of networks, dynamical or not.

In Section 2, a precise definition as well as notation are given. In the following Section 3 various applications are described. For example, phase transitions (and the inclusion of metastable phases) are defined in this formalism. Next we discuss the reconstruction of spaces—remarkably the OR can look like the original space. In Section 4 we deal with an ansatz not supported by theorems, but which occasionally works. This comes up when the spectrum of the transition probabilities has a non-zero imaginary part in its large eigenvalues. A final section summarizes the previous material.

There is some new material in this article, although it is mostly a brief review. Section 3.6, Appendix A, and Figure 4 in Section 3.1 and the discussion in the text are all published for the first time. The examples in Section 4 are also new.

## 2. Abstract Definition of the OR

Let *X* be a space having *N* elements. These elements are typically coarse grains of some finer dynamics, for example Hamiltonian dynamics, which, because of the coarse graining introduce a probabilistic aspect to time evolution. However, in many applications this view is too restrictive and dissipative or intrinsically probabilistic dynamics is contemplated. (There are even extentions to quantum mechanics [4].)

On the space *X* a *stochastic* dynamics is defined, i.e., for two elements x,y∈X there is a probability given that *y* goes to *x*:

(1) $$R_{xy}=\mathrm{Pr}\left(x\leftarrow y\right)\;.$$

Several points should be noted in this definition. First, the notation *R* has been introduced for the generator of the stochastic dynamics. (Sometimes this will be written $R_{xy}$, as above, sometimes R(x,y).) Second (and here other authors may differ) the arrow in Equation (1) points from right to left  [5]. Finally, there is an implicit time interval during which each step is taken, that is, each application of *R* is assumed to take the same time, say τ. In most situations τ will be taken to be 1, but for going to continuum dynamics (and the “master equation”) it will be useful to let τ→0.

As a result of *R*’s probabilistic nature the state (the element x∈X) will not, in general, be definitely known and the most one can give is probabilities. That is, there will be a function p(x,t) giving the probability that at time-*t* the system is in state *x*. Of course since the system is always *somewhere*, for each *t*, $\sum_{x\in X}p\left(x,t\right)=1$. Finally, we can give the relation between p(x,t) and p(x,t+1), namely

(2) $$p\left(x,t+1\right)=\sum_{y\in X}\mathrm{Pr}\left(x\leftarrow y\right)p\left(y,t\right)=\sum_{y}R_{xy}p\left(y,t\right)\;.$$

*R* is thus the generator of time evolution, and it was to make Equation (2) look good that the right-to-left convention for *R* was chosen.

One property of *R* is immediately evident: $\sum_{x}R_{xy}=1$, from any initial point *y* you have to go somewhere, and since X={x} is exhaustive the sum must give 1.

A matrix having non-negative elements and whose column sums are all 1 is called *stochastic*, so that our evolution matrix is stochastic. Stochastic matrices have an important spectral property: all eigenvalues are on or inside the unit circle. Proof can be found in [6] and other places.

A property that I’ll often assume is *reducibility*. Abstractly this means that there is no permutation operator, *P* such that $P^{T}RP$ has a lower left non-trivial block that is zero. ($P^{T}$ is the transpose of *P*.) If *N* (the cardinality of *X*) is small enough there is an easy computational way to find out if *R* is reducible: add the identity (I) to *R* and replace every non-zero element of R+I by 1 (calling the result *M*). You now have a matrix, *M*, of ones and (possibly) zeros. Compute $M^{N-1}$, and if there are no zero elements of this matrix then the original *R* is irreducible; otherwise it is reducible (see [7]). In other words, to be irreducible every state must be able to be reached from any other state. Under these circumstances the leading right eigenvector is unique. Call it $p_{0}\left(x\right)$. Then $p_{0}\left(x\right)>0$ for every *x* and it satisfies $Rp_{0}=p_{0}$, so it is the stationary probability distribution (often called the stationary *state*, which one could confuse with elements of *X*, but that is what people call it). By the way, it was clear that there would be a right eigenvector of *R* with eigenvalue 1, since we already had a left eigenvector with that property. That left eigenvector is all 1’s, i.e., we define $A_{0}\left(x\right)\equiv 1$ (for all x∈X) and note that the condition on *R* that all column sums be one, can be written $A_{0}=A_{0}R$, confirming that the eigenvalue is 1.

Call the eigenvalues $\left\{\lambda_{k}\right\}$ with $\lambda_{0}=1$ and the rest ordered by descending absolute value. Thus $\lambda_{0}\geq |\lambda_{1}\left|\geq \right|\lambda_{2}|\geq \ldots$. It can happen that there are fewer than *N* eigenvectors (Jordan forms, [8]), but that situation will not be considered here. This also imposes an order on the left and right eigenvectors. Call the right eigenvectors $p_{k}$ and the left eigenvectors $A_{k}$, for k=0,1,…,N−1. The left and right eigenvectors may be different although you still have $\sum_{x}A_{j}^{*}\left(x\right)p_{k}\left(x\right)=\delta_{jk}$, the Kronecker delta. Complex eigenvalues are put in pairs, so that if $\lambda_{k}$ is the first complex eigenvalue, then $\lambda_{k+1}=\lambda_{k}^{*}$, and so forth. (Complications can arise if the complex eigenvalues are also degenerate, but this will not be considered here.)

For complex eigenvalues not only do the eigenvalues $\lambda_{k}$ and $\lambda_{k+1}$ come in pairs, but so do the eigenvectors: thus $A_{k}^{*}=A_{k+1}$. This means that for *k* and (k+1) one can replace the true eigenvectors by ${\tilde{A}}_{k}=ℜ\left(A_{k}\right)$ and ${\tilde{A}}_{k+1}=ℑ\left(A_{k}\right)$. One can then define the “tilded” vectors that are real to replace the original eigenvectors and one has a full set $\left\{{\tilde{A}}_{k}\right\}$.

It is now possible to state what the OR is, although motivation and explanation of the word “observable” comes from the examples. Pick an integer *m*, with m≤N, and consider for each x∈X the *m*-component vector, $\left(A_{1}\left(x\right),A_{2}\left(x\right),\ldots ,A_{m}\left(x\right)\right)\in R^{m}$. This amounts to an embedding of the original space, *X*, in Euclidean *m*-space. (Or one could use the version with tildes, i.e., *A* replaced by $\tilde{A}$. See Section 4 for more on this subject.)

The *current*, an auxiliary quantity, can also be defined for a general stochastic process. For the stationary probability distribution $p_{0}$ we define

(3) $$J\left(x,y\right)\equiv R\left(x,y\right)p_{0}\left(y\right)-R\left(y,x\right)p_{0}\left(x\right)\;.$$

There is no summation convention above: *J* is defined for each *x* and *y*. Moreover, *J* satisfies Kirchoff’s rules and the sum of all currents at each point is zero.

*Detailed balance* is the situation in which all currents are zero. In this case all eigenvalues of *R* are real. This is easy to see, since detailed balance implies that the matrix $C_{xy}\equiv \frac{1}{\sqrt{p_{{}_{0}}\left(x\right)}}R\left(x,y\right)\sqrt{p_{0}\left(y\right)}$ is symmetric and since it is real, also Hermitian. This implies that all eigenvalues of *C* are real. Moreover, *C* has the same eigenvalues as *R*, with the right eigenvectors of *R* given as $\sqrt{p_{0}}$ times the eigenvectors of *C*.

Another feature that applies to all non-Jordan form matrices is the spectral expansion. One can write the matrix *R* in terms of its eigenvalues and left and right eigenvectors

(4) $$R\left(x,y\right)=\sum_{k=0}^{N-1}\lambda_{k}p_{k}\left(x\right)A_{k}\left(y\right)\;.$$

(Sometimes this is written $R\left(x,y\right)=\sum_{k=0}^{N-1}\lambda_{k}\left|k,x\rangle \langle k,y\right|$, although the bra-ket notation might obscure the fact that left and right eigenvectors can differ.)

## 3. Examples

We flesh out the abstract definition of the previous section with examples. The first set, dealing with phase transitions, is what motivated the original work, but the others followed with surprising relevance.

### 3.1. Phase Transitions

In the usual definition of phase transitions—a breakdown in analyticity—there is no such thing as supercooled water. For example, Isakov [9] proved that you cannot analytically continue the Ising model to a metastable phase (magnetization and magnetic field opposite to one another). However, if you look in handbooks [10] you can find the density of water at −2C. The problem is that the usual definition does not deal with non-uniform approximations. Once you let the number of particles or the volume go to infinity—which you must do to get a breakdown in analyticity—*somewhere* in this vast volume a critical droplet [11] will form and you no longer have a pure *metastable* phase. However, for real water in finite volumes, even macroscopic volumes, a critical droplet can take a long time—aeons—to form (for water you have to go to −30C to get short survival times and to about −40C for supercooling to cease [12]. This means that the time for formation of a critical droplet can be larger (in appropriate units) than the system size, and non-uniform limits are called for.

However, in stochastic dynamics a completely different definition is possible. As mentioned earlier, there is always an eigenvalue 1, and (for irreducible matrices) it is unique. What about the next few? A sign of a first order transition between two phases is if the next eigenvalue ($\lambda_{1}$) is very close to 1 and all others are much smaller. This applies also to metastable phases. Moreover the left eigenvector $A_{1}$ is nearly constant on states in *X* belonging to each phase, making its value a discriminator between the two phases; hence the term “observable.” We will see shortly why all this is true.

**Figure 1:**
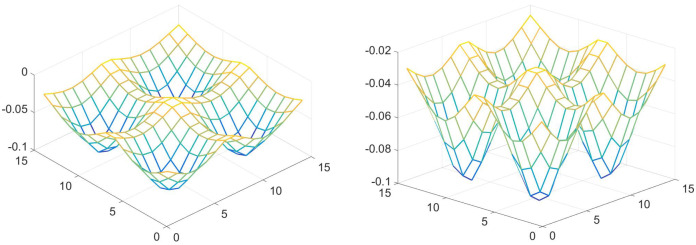
Two views of the same potential surface. The random walk prefers to move downhill, but occasionally (due to a finite temperature) will go to greater values of the potential.

It is simpler though to start with a Brownian motion example, namely a random walk on a potential surface. Take as the potential a surface with four minima, as in Figure 1. The space *X* is the 15-by-15 array of points on which the walk takes place. (So *R* is 225-by-225.) The stochastic process usually goes downhill (to lower values of the potential), but with some probability (dependent on the temperature) will go uphill. Step sizes are one and periodic boundary conditions are used. For low temperature it is clear what the result will be. The walker stays in one minimum for a long time, then every once in a while will move to one of the others (usually not crossing the center), where it also stays a long time.

**Figure 2:**
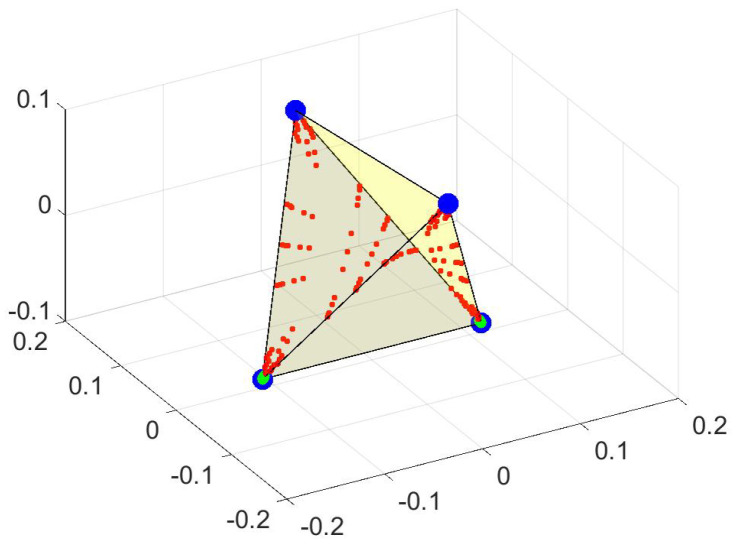
The OR for a random walk at low temperature in the 4-minima potential. This is a plot of the three first non-trivial left eigenvectors of the matrix *R* of transition probabilities, i.e., $A_{1}\left(x\right)$ vs. $A_{2}\left(x\right)$ vs. $A_{3}\left(x\right)$ for each x∈X= space of positions. These are the three axes of the figure. For each x∈X there is a certain probability of being found in the stationary state ($p_{0}\left(x\right)$). Different symbols are used for different categories of points, *x*. The largest probabilities are given the largest symbols, and these coincide with the extremal points (vertices) of the tetrahedron. Other points have smaller weight (values of $p_{0}\left(x\right)$) and are given smaller symbols (mostly red at this temperature, but some intermediate sizes, with green symbols, almost at the vertices). The convex hull of the points, which is the pictured tetrahedron, is shown as a guide to the eye. See the main text for further explanation.

The matrix *R* implements the rules just stated and can be diagonalized to yield eigenvalues and left eigenvectors. What is important is how close the eigenvalues are to 1, and for the example given the first five differences are $1-\lambda_{k}=\left(0.000429,0.000429,0.000794,0.1268,0.1284\right)$ (for k=1,…,5). Note the increase in $1-\lambda_{k}$ at k=4. As we shall see, this suggests a 3-dimensional plot of the first 3 (non-trivial) left eigenvectors. This is shown in Figure 2.

This is a perfect tetrahedron with the points having the largest probability shown with the largest markers. These correspond to the minima of the potential. Points near them have slightly lower probability while the peaks in Figure 1, while not zero, have very small probability. (For the example given, each of the minima has probability 0.07, while the peak in the center has probability $8\times 10^{-6}$.)

**Figure 3:**
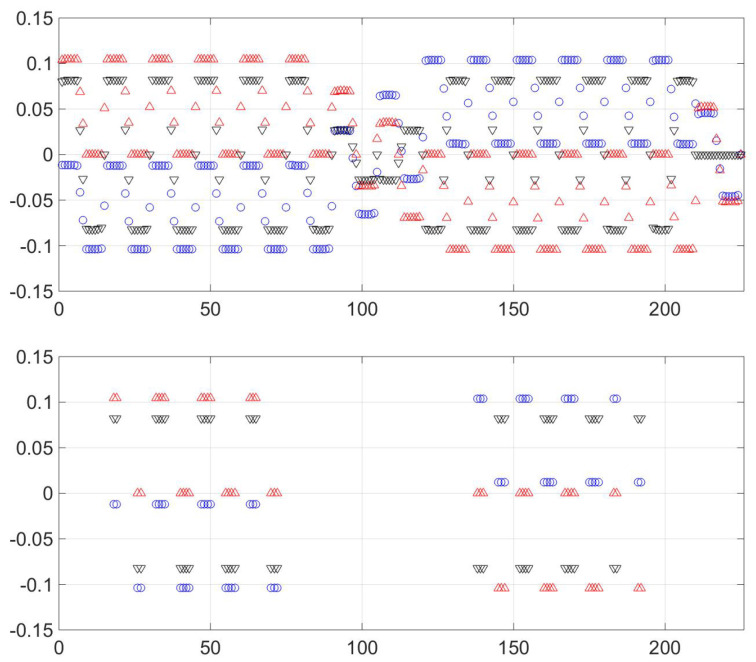
The first three left eigenvectors, pictured as blue circles, black downward pointing triangles and red upward pointing triangles. The upper figure shows all points, the lower shows only those with $p_{0}$ greater than $6\times 10^{-8}$. The *x*-axis is a number assigned to each of the 225 points and can be considered arbitrary. As can be seen—also in Table 1—only 10 values of the left eigenvectors have significant weight. This allows each phase to be characterized by the unique *A* values that characterize that phase.

The next pair of figures (Figure 3) require a bit of analysis, but lie behind the designation “observable representation” (OR). The upper figure shows the values of the three first (non-trivial) eigenvectors, and it is a mess. (The temperature is lower than for Figure 2 to accentuate features.) However, confining attention to only the “big” probability states, which in this case is all those greater than $6\times 10^{-8}$, one has the lower graph. Remarkably, each eigenvector only takes a few values, and any triplet of values characterizes a minimum (or a phase). This is shown in Table 1. That table is like a code. Each eigenvector is nearly constant on a phase, but each phase has a unique characterization, using those constants. For this reason the leading left eigenvectors are called the “observables.”

Note that there are points that are not at the vertices of the tetrahedron. These points have low probability and are not part of any phase. However, they have another interesting property. Suppose you give their *barycentric* coordinates (see Appendix B) with respect to the extremum points, namely those points that form the vertices of the tetrahedron. Barycentric coordinates must add to 1. However, so do probabilities. In fact, the barycentric coordinate with respect to any given extremum (phase) is just the probability that a walker starting from the given point ends up in that particular phase. (This is a theorem. See below.)

**Figure 4:**
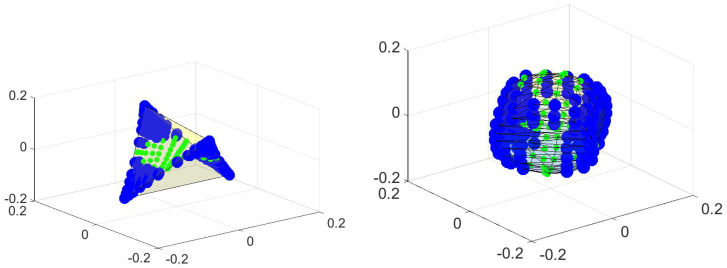
A sharp transition, whose explanation eludes me. On the left is the OR (a plot of $\left(A_{1}\left(x\right),A_{2}\left(x\right),A_{3}\left(x\right)\right)$ for all x∈X) for the same potential that is pictured in Figure 1, but at a higher temperature ($T_{\mathrm{tetrahedron}}\gg$ the temperature used in Figure 2). At this higher temperature I know of no reason for the points to form a tetrahedron, but they do. The probabilities of non-vertex points is higher (so the large symbols are far more common) but the shape of the convex hull is still a tetrahedron. Then, with a tiny fraction of temperature change ($10^{-9}$), the figure changes completely—roughly to a ball. The probabilities ($p_{0}$) of individual points do not change (significantly), but the OR becomes dramatically different.

There can also be surprises in the OR. By “surprise” I mean a property that I do not know how to prove. It turns out that the tetrahedral form of the OR is retained even for higher temperatures, even though the theorems that have been proved until now do not establish this. Then—suddenly—the figure becomes something altogether different. This is shown in Figure 4. The change in the figures represents a relative temperature change ($\left(T_{\mathrm{ball}}-T_{\mathrm{tetrahedron}}\right)/T_{\mathrm{tetrahedron}}$) of order $10^{-9}$. Up until $T_{\mathrm{tetrahedron}}$ the OR is recognizably a tetrahedron. From $T_{\mathrm{ball}}$ on it remains a ball. There is no recognizable change in the probability distribution nor in the spectrum (which no longer guarantees anything), but the change takes place in as short a temperature interval as I have patience to compute.

Phase transitions, whether between stable or metastable phases, have the same properties. The general notion follows from the eigenvalues of the matrix, *R*, of transition probabilities. The criterion is that the first few non-trivial eigenvalues must be close to 1, while those following them drop off quickly. (For now I consider the case of detailed balance, so all eigenvalues are real.) Suppose $1-\lambda_{k}\ll 1$ for k=1,…,n while $1-\lambda_{k}$ for k>n is much larger. Then the OR is a simplex of n+1 extrema in Euclidean *n*-space. Moreover, points deeply in the interior of the simplex (having barycentric coordinates with no nearly-1 component) have much lower probability ($p_{0}$ is small for those points). A way to look at this is to use the spectral expansion. Let *T* (a time) be sufficiently large that $\lambda_{k}^{T}$ is small for k>n but $\lambda_{k}^{T}$ is still close to 1 for k≤n. Then

(5) $$R^{T}\left(x,y\right)=\sum_{k=0}^{N-1}\lambda_{k}^{T}p_{k}\left(x\right)A_{k}\left(y\right)\approx \sum_{k=0}^{n}\lambda_{k}^{T}p_{k}\left(x\right)A_{k}\left(y\right)\approx \sum_{k=0}^{n}p_{k}\left(x\right)A_{k}\left(y\right)\;.$$

On this time scale the dynamics is fully equilibrated within each phase, and all that is left is the hopping between phases.

A proof of the simplex property is a bit complicated and for a full proof the reader is referred to [13]. (In that paper “*m*” is what is here called n+1.) For n=1 the proof is simpler although even in this low dimension some preparation is needed. First, $\sum_{x}A_{1}\left(x\right)p_{0}\left(x\right)=0$ by the basic orthogonality relation. Since (as usual) we assume irreducibility, we know that $p_{0}\left(x\right)$ is strictly positive. It follows that there is a positive maximum value of $A_{1}$, call it $A_{M}>0$ and (one of) the point(s) where it takes that value $x_{M}$. Similarly there is a negative minimum value, call it $A_{m}<0$ and define $x_{m}$ analogously. Now take as your initial point $x_{M}$. In other words $p\left(x,0\right)=\delta_{x,x_{M}}$. Evolve it for *T* time steps. Clearly almost all points remain in the relevant phase, which follows from the spectral expansion. What we do know is that

(6) $$A_{M}\lambda_{1}^{T}=\sum A_{1}\left(x\right)R^{T}\left(x,y\right)\delta_{y,x_{M}}\;,$$

since each application of *R* to the left leaves $A_{1}$ unchanged except for building up factors of $\lambda_{1}$, and $A_{1}\left(x_{M}\right)=A_{M}$ by definition. Call $\sum_{y}R^{T}\left(x,y\right)\delta_{y,x_{M}}$, which is the probability distribution after *T* time steps starting from $x_{M}$, $p_{M}\left(x,T\right)$. Similarly the same can be said for $x_{m}$ and its corresponding $p_{m}$. It follows that

(7) $$\lambda_{1}^{T}=\sum \frac{A_{1}\left(x\right)}{A_{M}}p_{M}\left(x,T\right)=\sum \frac{A_{1}\left(x\right)}{A_{m}}p_{m}\left(x,T\right)\;,$$

From the equalities $1=\sum_{x}p_{M}\left(x,T\right)=\sum_{x}p_{m}\left(x,T\right)$ we subtract Equation (7) to obtain

(8) $$1-\lambda_{1}^{T}=\sum \left[1-\frac{A_{1}\left(x\right)}{A_{M}}\right]p_{M}\left(x,T\right)=\sum \left[1-\frac{A_{1}\left(x\right)}{A_{m}}\right]p_{m}\left(x,T\right)\;,$$

The quantity on the left is nearly zero. The contribution from eigenvalues beyond k=1 is also negligible. It follows that to get the sums to be zero one or the other term in the respective products must be near zero. In other words, if $p_{M}\left(x,T\right)$ is not zero, i.e., if you are the phase associated with $x_{M}$ then $A_{1}\left(x\right)$ is going to be very close to $A_{M}$. The same is true for $A_{m}$; both are (nearly) constant on their respective phases.

With this result we are able to define phases. As usual there is a certain arbitrariness involved. Suppose we pick an *a* and define the phase $P_{\mu }$ to mean $|A_{1}\left(x\right)-A_{\mu }|<a$ (where μ is either *M* or *m*). The quantity *a* is small, but not too small, since as we shall see it appears in a significant denominator.

Our next goal is to establish that there is little probability outside the phases; that is $\sum_{x\notin \cup P_{\mu }}p_{0}\left(x\right)$ is small. Define $P_{M}^{\prime }$ to be the complement of $P_{M}$. Then

(9) $$\sum_{x\in P_{M}^{\prime }}p_{M}^{T}\left(x\right)<\frac{1}{a}\sum_{x\in P_{M}^{\prime }}p_{M}^{T}\left[1-\frac{A_{1}\left(x\right)}{A_{M}}\right]<\frac{1-\lambda_{1}^{T}}{a}\;.$$

And since $1=\sum_{x\in X}p_{M}^{T}\left(x\right)=\sum_{x\in P_{M}}p_{M}^{T}\left(x\right)+\sum_{x\in P_{M}^{\prime }}p_{M}^{T}\left(x\right)$ it follows that

(10) $$\sum_{x\in P_{M}}p_{M}^{T}\left(x\right)>1-\frac{1-\lambda_{1}^{T}}{a}\;.$$

The same relations hold with *m* (minimum) replacing *M* (maximum). Note that $\lambda_{1}$ may not be all that close to unity in some applications (for example in Figure 4) and (relatively) high probability *x*′s may occur at some distance from the extremum.

We next show that barycentric coordinates can serve as probabilities. For arbitrary *y* we look at $A_{1}\left(y\right)$. The fundamental equation for left eigenvectors can be written

(11) $$\lambda_{1}^{T}A_{1}\left(y\right)=\sum_{\mu }\sum_{x\in P_{\mu }}A_{1}\left(x\right)R^{T}\left(x,y\right)+\sum_{x\notin \cup P_{\mu }}A_{1}\left(x\right)R^{T}\left(x,y\right)\;.$$

At this point we need to give some normalization to $A_{1}$, since until now the only demand was $\sum_{x}A_{1}^{*}p_{k}=\delta_{1k}$. There are two natural normalizations. One can set $\max_{x}\left|A_{k}\right|=1$ for all *k*, and this is the normalization we here adopt. A second normalization, particularly when detailed balance obtains (which is not demanded for what we now prove) is L${\;}^{2}$ normalization, which will be described in detail in the next subsection. Either way we have $|A_{1}\left(x\right)|\leq 1$ and therefore

(12) $$\left|\sum_{x\notin \cup P_{\mu }}A_{1}\left(x\right)R^{T}\left(x,y\right)\right|\leq \sum_{x\notin \cup P_{\mu }}R^{T}\left(x,y\right)\leq \frac{1-\lambda_{1}^{T}}{a}\;.$$

Next $A_{1}$ within a given phase is replaced by $A_{\mu }$ for that phase with an error of order *a*. It follows that

(13) $$\lambda_{1}^{T}A_{1}\left(y\right)=\sum_{\mu }A_{\mu }\sum_{x\in P_{\mu }}R^{T}\left(x,y\right)+O\left(a\right)+O\left((1-\lambda_{1}^{T})/a\right)\;.$$

Two points should be noted about this equation. First, up to order $1-\lambda_{1}^{T}$, $\lambda_{1}^{T}$ is simply 1, and this factor already appears on the right hand side, divided by *a*, a number equal to or less than one. So set the $\lambda^{T}$ on the left to one. Consider the sum $\sum_{x\in P_{\mu }}R^{T}\left(x,y\right)$: it is just the probability that a point starting at *y* ends up in phase μ. We give it a name: $q_{\mu }\left(y\right)=\sum_{x\in P_{\mu }}R^{T}\left(x,y\right)$. Equation (13) becomes

(14) $$A_{1}\left(y\right)=\sum_{\mu }A_{\mu }q_{\mu }\left(y\right)+O\left(a\right)+O\left(\left(1-\lambda_{1}^{T}\right)/a\right)\;.$$

Now $A_{1}\left(y\right)$ is the position of the point *y* in the (one-dimensional) OR. What Equation (14) says is that up to $O\left(a\right)+O(\left(1-\lambda_{1}^{T}\right)/a)$, $A_{1}\left(y\right)$ and be expressed as a sum of the extremal points, $\left\{A_{\mu }\right\}$; that is, the quantities $q_{\mu }\left(y\right)$ are *barycentric* coordinates. Or to restate it, to a(n often) good approximation, the probability to enter one or another phase is given by the barycentric coordinates in the OR.

As for the usual OR these results generalize to a multidimensional OR. See [13] for details. To summarize: When there are *m* eigenvalues that are real and close to 1, with the other (N−m−1) much smaller in absolute value, then the OR is a simplex. Phases are defined through proximity to the extrema of the simplex and the barycentric coordinates of points within the simplex are the probabilities that starting from that point you will end in one or another phase. The proof relied on the fact that there is an intermediate time at which points within a phase have come to equilibrium, but the overall distribution of phase occupation will depend on the initial conditions.

### 3.2. Reconstructing Spaces 

Now consider the opposite case: the eigenvalues crowd around 1; they do not drop off suddenly. Under these conditions you do not expect a simplex nor weights (values of $p_{0}$) that are concentrated in phases; in fact there may not be phases.

**Figure 5:**
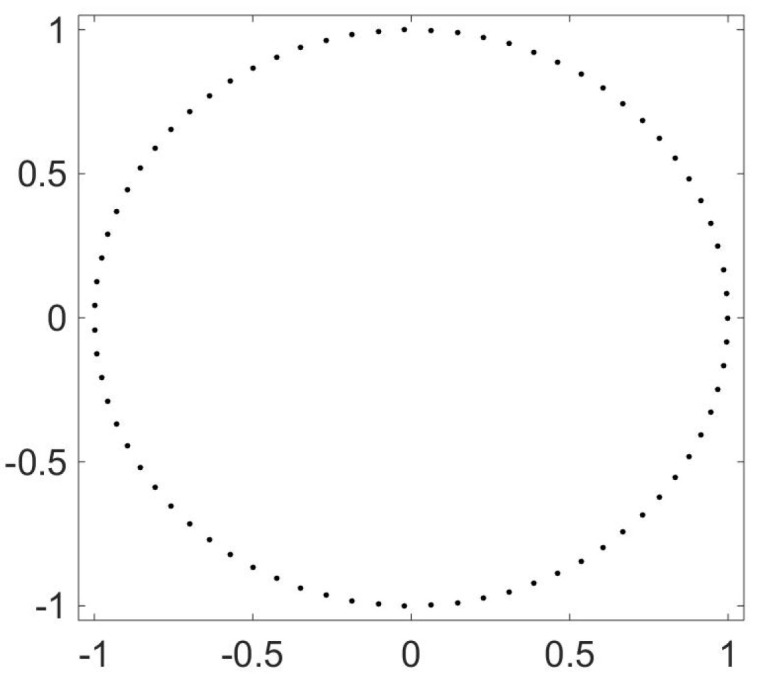
OR for a random walk on a circle of 100 points. The random walk has probability 1/2 of taking a step of size 1 in either direction on a circle. The figure though is not a plot of the original circle; rather it is the values taken by $\left(A_{1}\left(x\right),A_{2}\left(x\right)\right)$ for each x∈X, where *X* is the discrete set of positions on a circle.

Brownian motion on various configurations without a potential [14] is an example of this situation. Consider Brownian motion on *N* points arrayed in a circle. The transition probabilities for this can be taken to be all 1/2′s and zeros. The 1/2′s are just above and just below the diagonal, as well as in positions (N,1) and (1,N). This matrix can be diagonalized exactly and its eigenvalues are 1,cos(2π/N),sin(2π/N),⋯,cos(2πk/N),sin(2πk/N),…. The first two non-trivial (i.e., not all ones) left eigenvectors can be taken to be cos(2π/N) and sin(2π/N). Now the punch line: plot $A_{2}$ versus $A_{1}$ and you get Figure 5.

**Figure 6:**
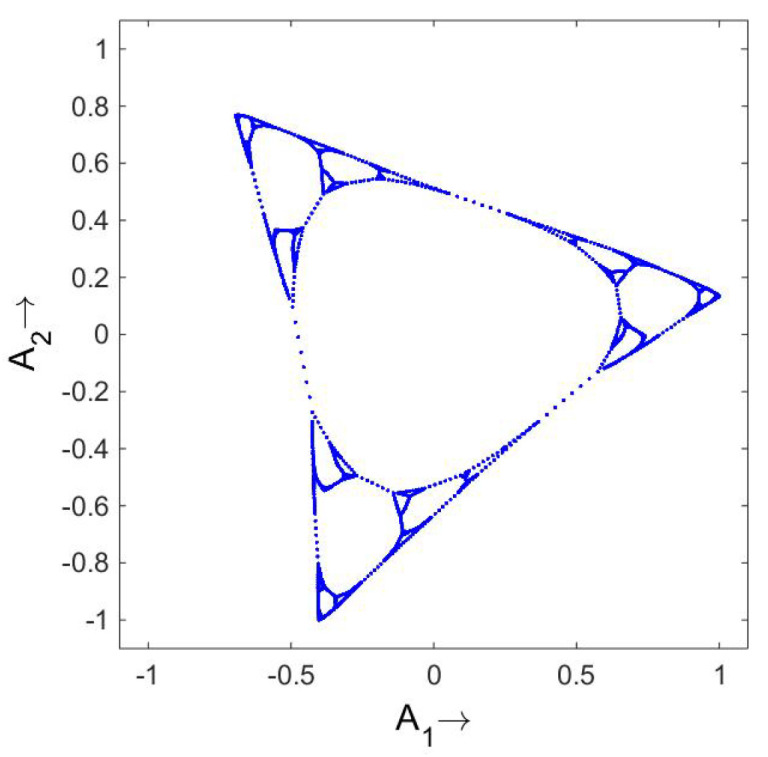
The OR for a random walk on a fractal. In this case the space *X* is a discrete representation of a fractal. When travelling on a straight portion of the fractal the walker has probability 1/2 of going in either direction. When the walker comes to a split there would be three choices, go back or go to one of the branches of the fractal. These are each given probability 1/3. As in the previous figure, what is illustrated is *not* the original fractal (*X*), but the OR, a plot of $\left(A_{1}\left(x\right),A_{2}\left(x\right)\right)$ for each x∈X.

**Figure 7:**
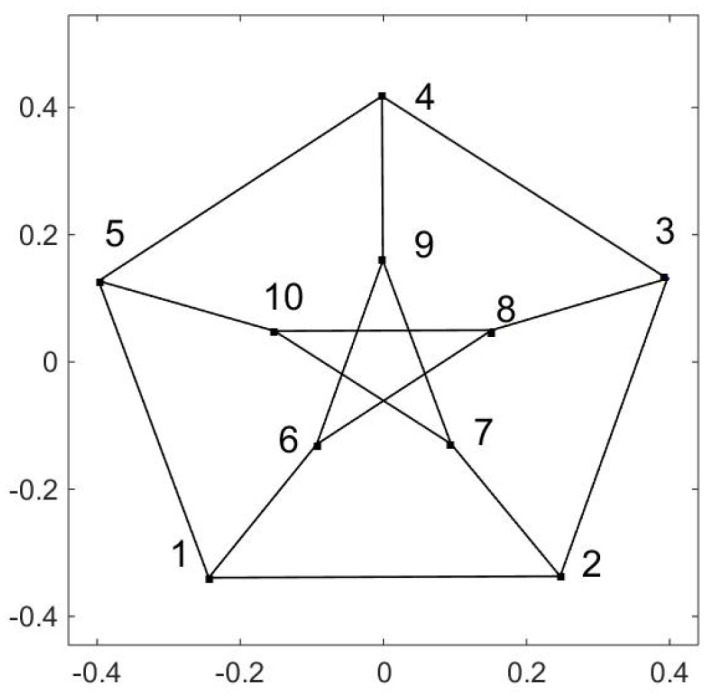
The Petersen graph. Note that there is no intersection in the interior pentagon; e.g., the line 6–8 does *not* touch the line 7–10. This graph cannot be embedded in a plane.

**Figure 8:**
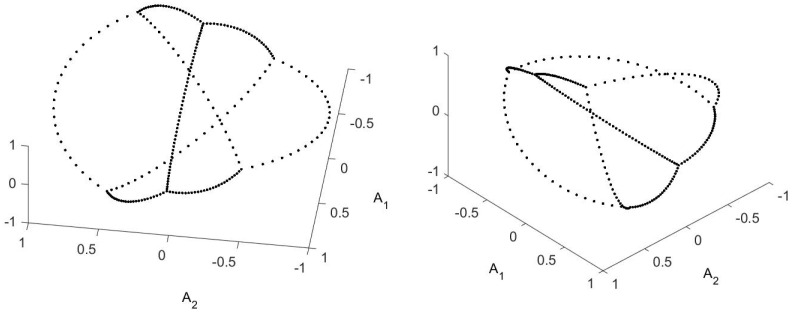
The OR for the Petersen graph. Two views are presented to emphasize that the OR also has the property that it needs three dimensions for an embedding.

How about a random walk on a figure Y; that is, the walker can get to the ends of the segments (no periodic boundary conditions) but in the middle there is one place where there is a choice of which branch to go on. I will not give a figure, but the OR is a Y. This continues to more complicated figures. As an extreme example, I used the computer to create a random walk on a fractal built of triangles. (This was computer generated so the fractal had 5 levels instead of going on indefinitely.) What is illustrated in Figure 6 is *not* the original fractal, but the OR that results from the stochastic matrix for a walk on the fractal. The eigenfunctions themselves (see [14]) give every appearance of being a mess, but somehow they conspire to produce the given figure. A second example of the power of this method is the Petersen graph. This is a graph with 10 vertices in which each vertex has 3 edges emerging from it. It has the property that *it cannot be embedded in a plane.* See Figure 7. It is interesting to see what the standard prescription for the OR does to this. The random walk again goes from vertex to vertex. Each line has several steps to make the connections visible. From the pair of graphs in Figure 8 it is clear that *three* dimensions are required in order to embed this figure.

**Figure 9:**
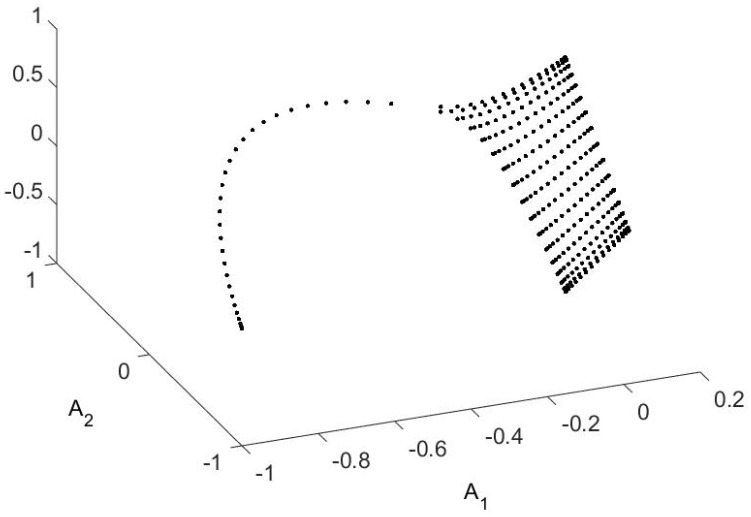
OR for a random walk on a square with “one-dimensional” path attached to one corner.

This works in higher dimension and even in “mixed dimension” (although strictly speaking we are always in dimension zero, since our space *X* is finite). As one illustration we show a random walk which is mostly on a square, but which can wander off onto a line. As in previous examples, what is shown in Figure 9 is the OR, not the original line plus square—although they look the same.

For other examples see [14].

### 3.3. Rationale 

In general, proximity in the OR reflects dynamical proximity. This was seen for phase transitions, for all sorts of Brownian motion and through the barycentric relation, which does not require detailed balance. On the other hand, if there is detailed balance a precise relation can be found.

We first generalize notation: Let $p_{y}\left(x,t\right)$ be the probability distribution for points that were at the point *y* at t=0. (This is a clear generalization of $p_{M}\left(x,t\right)$ or $p_{m}\left(x,t\right)$.) Clearly $p_{y}\left(x,t\right)=R^{t}\left(x,y\right)$.

For the case of detailed balance ($R\left(x,y\right)p_{0}\left(y\right)=R\left(y,x\right)p_{0}\left(x\right)$) we earlier saw that $\sqrt{p_{0}}$ played an important role. We now formalize that. Let σ be a diagonal *N*-by-*N* matrix with $\sigma \left(x,x\right)=\sqrt{p_{0}\left(x\right)}$. Then as observed earlier $C\equiv \sigma^{-1}R\sigma$ is symmetric and by virtue of its reality, Hermitian. As such, *C* has a complete set of eigenvalues and eigenvectors with

(15) $$C\left(x,y\right)=\sum \lambda_{k}\psi_{k}\left(x\right)\psi_{k}^{*}\left(y\right)\;,$$

with the same set of λ′s as for *R* and with $\left\{\psi_{k}\right\}$ orthonormal, i.e., $\sum \psi_{k}{\left(x\right)}^{*}\psi_{j}\left(x\right)=\delta_{kj}$. Since $p_{k}=\sigma \psi_{k}$ and $A_{k}=\sigma^{-1}\psi_{k}^{*}$ it is clear that this normalization of eigenvectors is different from setting the maximum of $|A_{k}|$ to be unity.

The probability distribution $p_{y}\left(x,t\right)$ can be thought of as a cloud of values; for example in the case of a phase transition if *t* is such that $\lambda_{1}^{t}$ is close to 1, but $|\lambda_{2}{|}^{t}$ is near zero, then $p_{y}\left(x,t\right)$ will either be an entire phase, or a weighted sum of the two of them, and will exclude (have small value at) points in between.

We define a distance between two such clouds

(16) $$D\left(x,y\right)=\sum_{u}\frac{\left|p_{x}\left(u,t\right)-p_{y}\left(u,t\right)\right|}{\sqrt{p_{0}\left(u\right)}}\;,$$

Note that

(17) $$\frac{p_{x}\left(u,t\right)}{\sqrt{p_{0}\left(u\right)}}=\frac{1}{\sqrt{p_{0}\left(u\right)}}{\left(\sigma C^{t}\sigma^{-1}\right)}_{ux}=\sum_{\alpha }\lambda_{\alpha }^{t}\psi_{\alpha }\left(u\right)\frac{\psi_{\alpha }^{†}\left(x\right)}{\sqrt{p_{0}\left(x\right)}}=\sum_{\alpha }\lambda_{\alpha }^{t}\psi_{\alpha }\left(u\right)A_{\alpha }\left(x\right)\;,$$

The function D(x,y) of Equation (16) is of the form of an L${\;}^{1}$ norm. The L${\;}^{1}$ norm is always equal to or greater than the L${\;}^{2}$ norm, and therefore

(18) $$D\left(x,y\right)\geq {\left(\sum_{u}\frac{\left|p_{x}\left(u,t\right)-p_{y}\left(u,t\right)\right|}{\sqrt{p_{0}\left(u\right)}}\right)}^{1/2}=\sqrt{\sum_{u}{\left|\sum_{\alpha }\psi_{\alpha }\left(u\right)\lambda_{\alpha }^{t}\left[A_{\alpha }\left(x\right)-A_{\alpha }\left(y\right)\right]\right|}^{2}}\;.$$

This expression is of the form ${\left[\sum_{u}\sum_{\alpha }\sum_{\alpha^{\prime }}\psi_{\alpha }\left(u\right)\psi_{\alpha^{\prime }}^{*}\left(u\right)c_{\alpha }c_{\alpha^{\prime }}^{*}\right]}^{1/2}$, which because of the orthonormality of the ψ′s is ${\left[\sum_{\alpha }{\left|c_{\alpha }\right|}^{2}\right]}^{1/2}$. It follows that

(19) $$D\left(x,y;t\right)\geq \sqrt{\sum_{\alpha }{\left|\lambda_{\alpha }\right|}^{t}{\left|A_{\alpha }\left(x\right)-A_{\alpha }\left(y\right)\right|}^{2}}\;.$$

The sum on the right can be truncated at the *m*th eigenvalue, preserving the direction of the inequality. Since the magnitudes of the eigenvalues decrease monotonically (with index) we obtain, finally,

(20) $$D\left(x,y;t\right)/|\lambda_{m}{|}^{t}\geq \sqrt{\sum_{\alpha =1}^{m}{\left|A_{\alpha }\left(x\right)-A_{\alpha }\left(y\right)\right|}^{2}}\;.$$

The quantity on the right is the distance in the OR. Thus, two points *x* and *y* which are adjacent dynamically, meaning their clouds overlap, will be spatially adjacent in the OR.

### 3.4. Spin Glass 

The spin glass has Hamiltonian $H=-\sum_{\langle ij\rangle }J_{ij}\sigma_{i}\sigma_{j}$ and has served as a model for many phenomena, from glass to the brain. The coupling constants, $\left\{J_{ij}\right\}$, are typically random but quenched (meaning they are fixed for each calculation); they are usually taken to be either ±1 or with values in a normal distribution. The spins, $\left\{\sigma_{i}\right\}$ are ±1. The pairs 〈ij〉 are usually nearest neighbors on a lattice of some sort. A model of the model is a mean field version known as the Sherrington-Kirkpatrick model (SK). The Hamiltonian is

(21) $$H=-\frac{1}{\sqrt{2N}}\sum J_{ij}\sigma_{i}\sigma_{j}\;,$$

where the sum is now over all *i* and *j* (different from each other). The states are now $x\equiv (\sigma_{1},\sigma_{2},\ldots ,\sigma_{N})$ and the energy is E(x)=H(x). The transition probability for *x* and $x^{\prime }$ differing by the value of a single spin is

(22) $$R\left(x^{\prime },x\right)=\left\{\begin{array}{cc} \frac{1}{N}\exp\left(-\frac{\left[E\left(x^{\prime }\right)-E\left(x\right)\right]}{T}\right) & \mathrm{if}\;E\left(x^{\prime }\right)>E\left(x\right)\\ \frac{1}{N} & \mathrm{otherwise} \end{array}\right.\;,$$

Then the diagonal of *R* is adjusted to make all column sums unity. This transition probability matrix guarantees that the stationary probability distribution is just $p_{0}\left(x\right)=\exp\left(-E\left(x\right)/T\right)/Z$ with $Z=\sum_{x\in X}\exp\left(-E\left(x\right)/T\right)$.

The SK model spin glass [15] offers a mixture of the previous situations. Yes, there are phases, but the spectrum of eigenvalues has many points near 1. This is because there are *many* phases, most of them metastable. The idea is that the system passes slowly through ever deeper (in energy) metastable phases on its way to the stationary probability distribution.

To diagonalize *R* you first need to fix a matrix, *J* (the set $\left\{J_{ij}\right\}$), of couplings; in this case each coupling was ±1. For *N* spins there are $2^{N}$ different states, so that *R* is a $2^{N}$-by-$2^{N}$ matrix. For the computer used (and the limited skill of the user) this meant that for most calculations *N* was 12 or 13. Nevertheless, insight into the coordinate space and the progression of metastable phases could be gained.

First, each $x=(\sigma_{1},\ldots ,\sigma_{N})$ would naturally be associated with a point on the unit *N*-cube, a not very helpful association. A measure of distance could be hamming distance or Euclidean distance, neither of which would describe the underlying dynamics, which also depends on the matrix *J*. With the OR there is an embedding that respects the dynamics, so that proximity means that one state can easily become the other.

**Figure 10:**
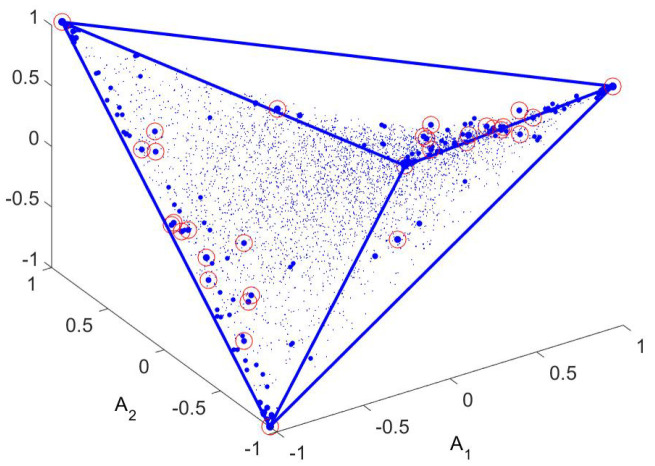
Symbol size increases with increasing $p_{0}$ (hence lower energy). Circles indicate local minima in energy. The convex hull (a tetrahedron) has been outlined. Many of the minima are vertices (extrema) in a higher dimension convex hull; this has been checked to dimension seven.

Having more than 4 phases means that three dimensions are not adequate to fully visualize the geometry. Nevertheless, one can find the convex hull in more than three dimensions and mark the relevant state (the vertices) on a 3-dimensional plot. (Actually, barring the possibility that this article will be published with a 3-dimensional printer, what you will see is a 2-dimensional plot of a 3-dimensional object.) For a particular choice of *J* and *T* this is shown in Figure 10.

**Figure 11:**
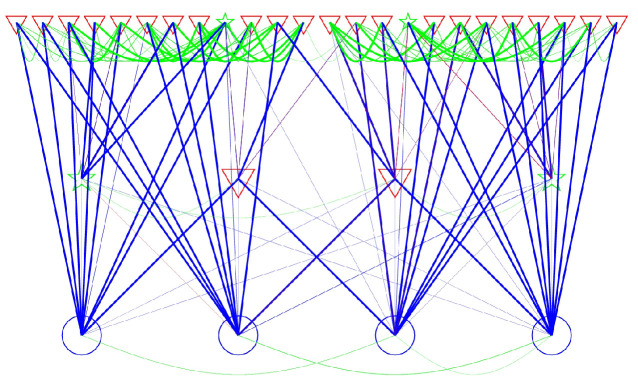
Transitions between phases. The phases are sorted (based on measured lifetimes) into 3 categories, long-, medium- and short-lived. On the lowest level are the longest-lived, etc. The symbol and color correspond to the amount of inflow. A phase’s left-right position follows the $A_{1}$ values of that phase’s nucleus among phases at the same lifetime level. Lines indicate transition probabilities, with line width a monotonic function of transition probability. Blue lines are flow from higher (shorter lifetime) levels to lower, red go oppositely, and green represents lateral transitions.

What does the OR tell you about the dynamics? It provides extrema and a distance scale. By the procedure mentioned earlier (cf. the paragraph between Equations (8) and (9)) this allows us to define phases. (Further verification is that for the example in Figure 10, 40% of the points lie outside all phases, and yet their *combined* probability is about $2\times 10^{-5}$). What about the flow between phases? For that it suffices to start a bunch of points in a phase and see where they end. This leads to an effective $\tilde{R}$ between phases. In this case there is an element of randomness due to the routes of various points from the phases. (You could also add the transition probabilities from the each point in a given phase.) The diagram of transitions (with the randomness) is illustrated in Figure 11.

The figure can be considered a test—or a reflection—of the hierarchical model. That is the picture of phases flowing into ever deeper metastable phases. Probabilistically there should be flow in both directions, but in a larger setup the flow to less likely phases would be quite small. In the illustration some of the wrong-direction flow happens to be large but this can be attributed to random paths. It is interesting though that, up to randomness, the OR distances (and resulting phases) reproduce the hierarchical model.

### 3.5. Foraging 

How do animals look for food? Lots of different strategies, all of them reasonably successful, or else the species would cease to exist. In recent work [16] Wosniack calculated foraging behavior for an animal that was far-ranging and with food sources that were renewable. She used exact positions (with machine precision), but when it came to analyze the data coarse graining was performed. However, at this stage all she had was a single path. It was long enough to come back to the same position several times, so it was a candidate for the method of Appendix A. (See that section for details.) Food sources were at particular positions in this (periodic) universe, and each food source gave an extremum in the OR. In the simulation she used 5 places where food could be found. This meant there would be 5 extrema. How to picture them?

**Figure 12:**
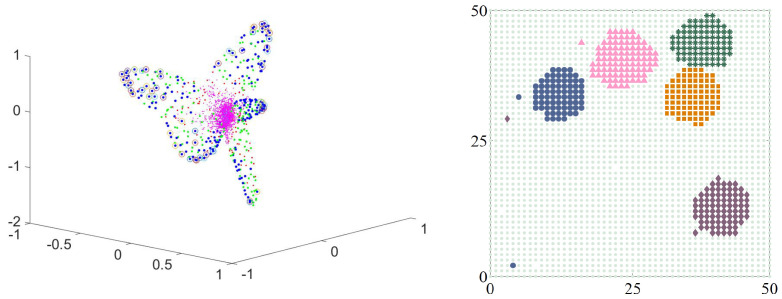
On the left is the OR for foraging with 5 extrema. It is possible to see that there are 5 extrema, but there isn’t much precision in their location. However, using k-means plus the mapping to the original two-dimensional space (of the forager) the locations of the food sources could be recovered (right hand figure).

The key was a mapping back to the original two-dimensional space. For details see the original article [16], but with this additional piece of information (and using k-means) the location of the food sources could be established. See Figure 12.

### 3.6. Complex Systems 

Complexity has yet to be defined, but people will agree that linguistics, ecology and protein networks can qualify. An example of an application to each field is given in [17]. Occasionally the embedding is easy to understand and has the appearance of one of those I’ve already dealt with. However, there are also situations where deductions require a bit of knowledge of the system. Even in our earlier examples if the number of extrema exceeded four, other tricks had to be used for visualization. Still, as one can see from the spin glass example, it is possible to find structure (e.g., hierarchical properties) in the system.

In the present article I’ll give an example of an OR that I do not understand. The results are significant, the system is arguably complex, but I do not understand what’s going on. Sometimes the OR brings surprises, and sometimes you (or I, anyway) do not know why the figure picks out special points.

I took the text of Melville’s novel, Moby Dick (“Call me Ishmael. …”). The question was if letter 337 was an “a” what would letter 338 be? In other words I got the probability of ordered pairs, what is most likely to follow each letter. To make things easily doable I made several simplifications. I got rid of commas and other markings such as quotation marks, changed semicolons to periods and all capital letters to lower case, so in the end there were only 28 characters, the Roman alphabet (sans accents) plus a period and a space. Then I counted all ordered pairs and these became the first version of the transition matrix. However, the columns sums were not all the same. Thus, if R(i,j)=const·Pr(letter #*j* goes to letter #i) then the sums, sum${}_{j}=\sum_{i}R\left(i,j\right)$, vary. Since all these numbers should be one, further measures must be taken—and they are not unique. In MATLAB${}^{{}^{\mathrm{TM}}}$ notation, you would have choices:

R=R/max(sum(R)); followed by R=R+diag(1-sum(R)); or

R = R*diag(1./sum(R)); and many others.

If you choose the first method, you divide *R* by the maximum of its column sums and make it up in the diagonal terms. In the second scheme you divide each column by its sum. I prefer the first method because for equilibrium systems it gives the Boltzmann distribution as it is stationary probability distribution. Also, in the present instance scheme #1 gives interesting results, Scheme #2, as far as I know, does not.

**Figure 13:**
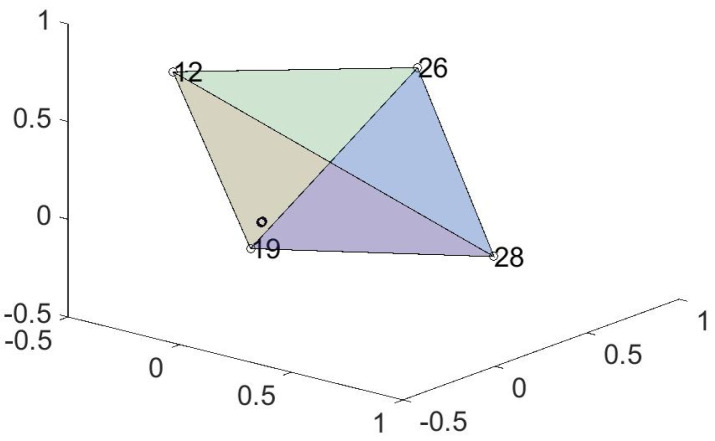
Letter pairings from the book, “Moby Dick,” with the transition matrix defined by R(k,j)=Pr(sybmoljisfollowdbysymbolk). The “symbols” are the 26 letters of the Roman alphabet, plus a period and space. The letters corresponding to the points 12, 19, 26 and 28 are j, q, x and z (the first two on the list are a space and a period).

Those “interesting results” are illustrated in Figure 13. You can see that there is a well-formed tetrahedron, with the extremal points indicated. All the other points are gathered in that fuzzy image near the vertex at point #19. Now the first few eigenvalues are 1,0.9972,0.9952,0.9947,0.9924,0.9618,0.9587) so there is no sudden dropoff after $\lambda_{3}=0.9947$, but there is still a tetrahedron. However, the real surprise comes when the identity of the vertices are revealed. They are the letters “jqxz” (not ordered). These are the most rare letters in the book. One can also look at the 4-dimensional convex hull formed by the first 4 non-trivial eigenvectors, and one gets “jkqvxz”, again rare letters. (There are 6 letters, rather than 5, because the convex hull is not a perfect simplex.) Why in 3 dimensions one gets a neat tetrahedron, and why the rare letters are at the vertices, I do not know. The OR is full of surprises, and this one has me baffled.

## 4. Currents and Complex Eigenvectors 

Current was defined in Section 2 as $J\left(x,y\right)\equiv R\left(x,y\right)p_{0}\left(y\right)-R\left(y,x\right)p_{0}\left(x\right)$ and it was there proved that if the current at all points in *X* is zero then detailed balance prevails and all eigenvalues of *R* are real. However, currents often do occur and eigenvalues can be complex. For example, in the results of Section 3.6 (Moby Dick) the first few eigenvalues are real, but the 9th and 10th (counting 1 as the zeroth, and numbered in decreasing magnitude) have non-zero imaginary parts, as do the pairs (21,22) and (24,25). Complex eigenvalues mean complex eigenvectors.

Remark:  The occurrence of non-zero current has been suggested [18] as a basis for evaluating complexity. The current has a topological structure and is thus richer than the assignment of a single number as a measure of complexity. Clearly all detailed balance systems are (by a definition based on currents) not complex, so that only non-equilibrium systems are candidates for complexity. However, a general definition has yet to be offered.

As currents increase (in any particular model), so does the imaginary part of the eigenvalues. In addition—in my experience—it also moves up the eigenvalues. That is, at first it is the smallest eigenvalues that have imaginary parts, but as the current increases larger and larger eigenvalues acquire imaginary parts. Finally this process reaches the eigenvectors (which have imaginary parts once the eigenvalues do) that we wish to plot; that is, the leading left eigenvectors are complex.

At this point, we are left without theorems. Barycentric coordinates, simplices, all those results only hold for real eigenvectors. What to do? Gaveau and I have managed some theorems about high powers of *R*, but have not yet found them to be practical. Also Nicholson et al. [19] have proposed another operator, call it *B*, with dynamics close to that of *R*, which does satisfy detailed balance [20]. However, the dynamics of *B* may be close, but in my opinion, not close enough. So the short answer to my earlier question, like much else in the use of the OR, is “try.” What happens if a left eigenvector that you plan to plot is complex, take its real and imaginary parts and plot them. This actually does not change the number of left eigenvectors plotted, since there is another left eigenvector that is the complex conjugate of the one you’re plotting. (In case that complex eigenvector is the last one to be plotted its real part is usually taken.) Sometimes this works to give interesting results, sometimes not.

**Figure 14:**
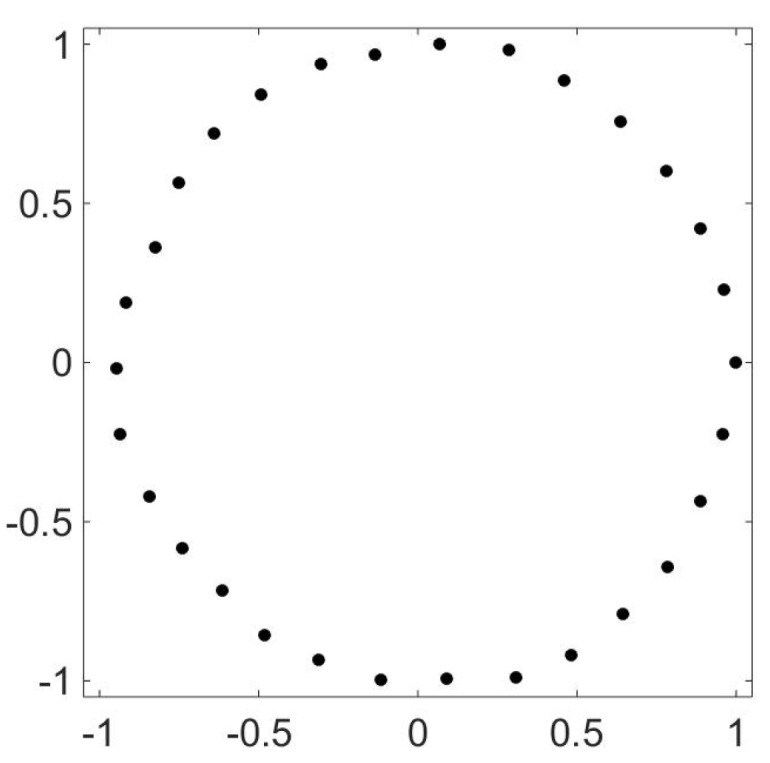
Modified OR for a permutation, which has complex roots. It is modified in the sense that instead of plotting real eigenvectors against each other, the plot is of the real and imaginary parts of the first eigenvector.

An example is the permutation operator. Except for a small amount of random noise that I impose (in order that $\lambda_{0}$ be the only eigenvalue of absolute value 1) this matrix takes 2 to 1, 3 to 2, …, and 1 to *N* (with N=30 in this case). This gives exactly the same representation as does the random walk on the circle, which has detailed balance. So taking real and imaginary parts can work in some cases. See Figure 14.

**Figure 15:**
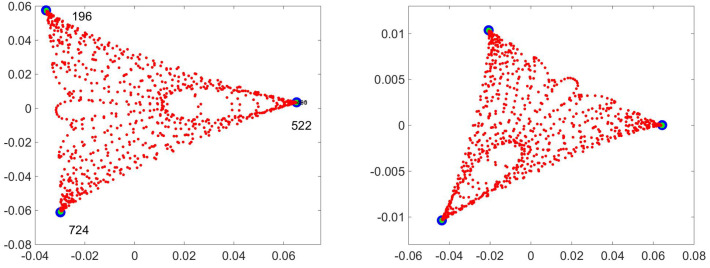
On the left is the OR for a three minima potential whose eigenvalues are real. On the right, I have added to the transition probability a travel shortcut between the minima, making the eigenvalues (and eigenvectors) complex. Up to re-orientation there is no difference between the figures.

Another case in which taking the real and imaginary part works is a walk in a potential. On the left of Figure 15 is the OR for a walk in a potential with 3 minima. For this walk [21] it is found that the extrema of the triangle are are the points numbered 196, 522 and 724 (the stochastic matrix is 961-by-961 ($=31^{2}$). In the right hand figure I’ve added a term that allows a travel shortcut between the minima, a matrix connecting the three extrema. This makes the eigenvalue complex and induces a current. The right hand figure of Figure 15 uses the real and imaginary parts of the first non-trivial eigenvector. As you can see, it is (essentially) the same diagram. This does not always happen, but in this case it did.

## 5. Summary and Conclusions 

The observable representation (OR) is a dynamics-respecting embedding of the space of states into Euclidean *m*-space, where typically *m* is two or three. A “state” is a recognizable configuration of a system, typically a coarse grain. “Dynamics respecting” means that typically the easier it is for a given state to become another state under the dynamics, the closer they will be in the OR. The original motivation for this work concerned metastable phases [22]. Conventional definitions of phase transitions involving analyticity fail when applied to metastable phases, leading Bernard Gaveau and myself to a different definition, one depending on the spectrum of the matrix of transition probabilities. The matrix, called *R* in the present article, plays a central role in the definition of the OR. Call its eigenvalues $\lambda_{0},\lambda_{1},\ldots$ in descending value (which may need further definition; see below). Then $\lambda_{0}=1$, and near degeneracy of $\lambda_{1}$ may be the hallmark of a second phase, i.e., there may be two collections of states of *X* representing two phases, one of which may be metastable. This can easily be generalized to more phases although embedding more than 4 phases may require more than three dimensions.

The original work on phases can be summarized as follows. If there are m+1 phases there need to be *m* eigenvalues nearly degenerate with 1, and $|\lambda_{m+1}|\ll \lambda_{m}$. The OR in this case becomes (to a good approximation) a simplex with the barycentric coordinates of internal points the probability that with that point as an initial value the system goes to one or another phase.

However, the OR turns out to have meaning well beyond the definition of phases. For a random walk without potential it can reproduce the space on which the walk takes place. If there are more extrema than can be imaged in three dimensions there can be ways to pick out the overall structure. In addition, there are surprises, for example in the linguistic analysis of the book, Moby Dick, for reasons unknown (to me) the OR is a tetrahedron with the rare letters the extrema.

Finally there is a special class of images for which we do not have theorems but which sometimes work. I refer to the situation when some of the large eigenvalues are complex. In this case I took the real and imaginary parts and a useful image emerged. The ordering of the eigenvalues is also affected and usually they are ordered by absolute value, with an arbitrary choice within one particular absolute value.

To summarize, if it is easy to do, do it. For some cases it is known that the OR gives useful information, for some case it manages to do so despite the absence of theorems. Various special cases have been studied, but for a stochastic process that does not fit in one of the categories studied, there may or may not be useful information in the plot.  

## Figures and Tables

**Table 1:** Values of the left eigenvalues on the principle points of the 225 ($=15^{2}$) of the space *X*. The phases are characterized by reading *down*. Thus phase 1, a vertex of the tetrahedron in Figure 2, is characterized by $A_{1}=-0.0118$, $A_{2}=0.105$ and $A_{3}=0.819$.

$A_{k}$, Symbol in Figure 3	Value on Phase 1	Value on Phase 2	Value on Phase 3	Value on Phase 4
1, circle	−0.0118	−0.104	0.104	0.0118
2, triangle, up	0.105	$3\times 10^{-5}$	$1\times 10^{-5}$	−0.105
3, triangle, down	0.819	−0.819	−0.819	0.0819

**Figure A1:**
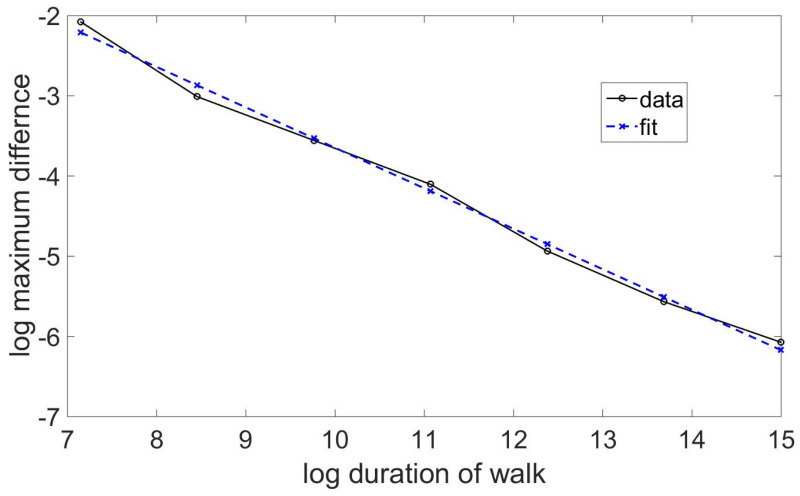
Dependence of $\max_{x}\left|R\left(x\right)-R_{\mathrm{orig}}\left(x\right)\right|$ on length of the path for a system without metastable states.

**Figure A2:**
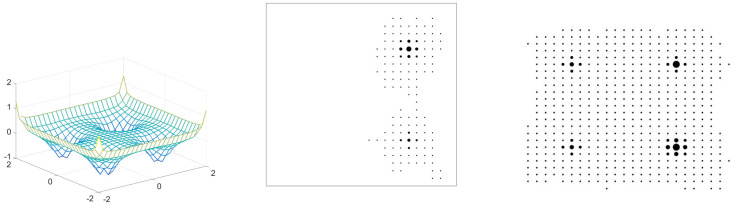
On the left is a potential with 4 minima, two of them slightly deeper than the others. The other two figures show the occupation: the size of a dot is proportional to the weight of the stationary distribution of the matrix *R* for the given path length. For the middle figure $T∼2\times 10^{5}$ and a boundary is shown to emphasize the regions *not* visited. For the image on the right much longer times are used.

## Appendix A. How to Handle (Some) Large Spaces

It often happens that a state space is large. For example in the foraging case, even coarse graining, still leaves a 50-by-50 state space. Thus “*X*” is of cardinality 2500 and would require a transition probability matrix that is 2500-by-2500. Or one could try to create an OR for the Jensen evolutionary process (“Tangled Nature,” see, e.g., [23]) where the state space can be infinite.

In practice it is generally easier to trace a particular path through the space than to write down an entire transition matrix. In this section I will describe how to use such a path to recover an approximation to the transition matrix. Generally it involves fewer points than the entire state space.

Suppose one is given a specific random walk, x(t), t=1,…,T. The nature of the space in which *x* takes its values is irrelevant, and we replace it by integers. Thus x(1) is just 1. If x(2) is different it is called 2; otherwise it is also 1. The total state space *of interest in the present construction* is the set of unique values taken by {x(t)|t=1,…,T}. The actual state space may in principle be infinite, but this is not a problem. Let the number of unique sites be called *N*, so that “*R*”, the set of approximate transition probabilities, is an N×N matrix.

Define an intermediate quantity S(a,b) to be the number of times (out of all T−1 time steps) that *b* goes to *a*, where *a* and *b* are integers (i.e., x(t)=b and x(t+1)=a). The column sums of *S* are of course not in general 1, but that is less serious than the fact that *S* may be reducible. A way to cure this is to introduce an artificial step: Let x(T+1)=x(1) and add this contribution to *S*.

As indicated in Section 3.6 there are various ways to make *S* stochastic. Let *s* be its column sums and $\tilde{s^{-1}}$ the diagonal matrix made from the inverses of *s*. In this section we multiply the matrix *S* by $\tilde{s^{-1}}$. (In MATLAB${}^{{}^{\mathrm{TM}}}$ notation the sum is sum(S) and the method used here is R=S*diag(1./sum(S)), where diag is the diagonal matrix whose (possibly) non-zero elements are the argument of diag.)

It must be emphasized that this is an uncontrolled approximation (at least as far as the author knows). It works when it works. What is shown in [24] is that sometimes it does work (as in the case of foraging) and sometimes it does not. The latter situation does not lead to publication, which may cause a biased view.

One way to see the efficacy of this strategy is to produce a path using a definite set of transition probabilities, to be called $R_{\mathrm{orig}}$. Then use the method described above to see how effective the recovery is. In [24] it is found that there are several major factors in the accuracy of the recovery. The first is *T*, the length of the path. A measure of accuracy is the largest (in absolute value) discrepancy, i.e., $\max_{x}\left|R\left(x\right)-R_{\mathrm{orig}}\left(x\right)\right|$, and this is found to decrease as $T^{-1/2}$. See Figure A1. Then there is the size of the matrix. Obviously the larger the matrix, the more steps are needed. However, if additional steps are given the discrepancy actually decreases. Roughly, for *N* up to N=2048 and the time growing linearly in *N*, the decrease in accuracy (for an *N*-by-*N* matrix) went roughly like Discrepancy$\;∼N^{-0.4}$. Finally, there is the possible existence of metastable states. The path x(t) may get stuck in one particular metastable state and not explore others, even for large *T*. This was tested in the potential illustrated in Figure A2 and the results of the procedure described above for various times (*T*) are also shown. Note that even for the shorter time, when two of the metastable states are not explored at all, the pictures of the states that *were* visited is fairly accurate.

## Appendix B. Barycentric Coordinates

Let $\left(y_{1},\ldots ,y_{n+1}\right)$ be the vertices of a simplex in *n*-dimensional Euclidean space. (Each $y_{k}$ is itself an *n*-vector.) Then a vector x contained in the simplex, which is convex, can be written

(B1) $$x=\sum_{k}\mu_{k}y_{k}\;,$$

with each $\mu_{k}\geq 0$ and $\sum_{k}\mu_{k}=1$. If a point lies outside the simplex it can still be written in the form (B1), but at least one of the $\mu_{k}$′s will be negative.

In one dimension, $y_{1}$ and $y_{2}$ are just points on a line and any other point can be written $x\;=\;\mu_{1}y_{1}\;+\;\mu_{2}y_{2}$ with $\mu_{k}=\frac{x-y_{k^{\prime }}}{y_{k}-y_{k^{\prime }}}$ and $k^{\prime }\neq k$. In the general case one can use $\mu_{n+1}=1-\sum_{1}^{n}\mu_{k}$ to obtain *n* equations in *n* unknowns (the remaining $\mu_{k}$′s) with non-vanishing matrix because the simplex has strictly positive volume in *n*-space.
